# Sex differences in adults with attention-deficit/hyperactivity disorder: A population-based study

**DOI:** 10.1192/j.eurpsy.2025.2441

**Published:** 2025-04-11

**Authors:** Ferran Mestres, Vanesa Richarte, Juan Jesús Crespín, Carla Torrent, Santiago Biel, Carolina Ramos, Pol Ibáñez, Laura Oltra-Arañó, Montse Corrales, Silvia Amoretti, Christian Fadeuilhe, Josep Antoni Ramos-Quiroga

**Affiliations:** 1 Department of Mental Health, Hospital Universitari Vall d’Hebron, Barcelona, Spain; 2 Group of Psychiatry, Mental Health and Addictions, Vall d’Hebron Research Institute (VHIR), Barcelona, Spain; 3 Biomedical Research Networking Cente for Mental Health Network (CIBERSAM), Instituto de Salud Carlos III (ISCIII), Barcelona, Spain; 4 Department of Psychiatry and Forensic Medicine, Universitat Autònoma de Barcelona, Barcelona, Spain; 5 Bipolar and Depressive Disorders Unit, Hospital Clínic de Barcelona; Institut de Neurociències (UBNeuro); Fundació Clínic-Institut d’Investigacions Biomèdiques August Pi I Sunyer (IDIBAPS), Barcelona, Spain; 6 Departament de Medicina, Facultat de Medicina i Ciències de la Salut, Universitat de Barcelona (UB), Barcelona, Spain

**Keywords:** ADHD subtypes, attention-deficit/hyperactivity disorder (ADHD), functional impairment, psychiatric comorbidities, sex differences

## Abstract

**Background:**

Attention-deficit/hyperactivity disorder (ADHD) is a neurodevelopmental disorder that often persists into adulthood, significantly impacting daily functioning and quality of life. Sex differences influence ADHD presentation, with females experiencing delayed diagnosis and distinct patterns of severity and comorbidities. Exploring these differences is essential for improving diagnostic accuracy and developing tailored interventions. This study examines ADHD severity, psychiatric comorbidities, and functional impairment by ADHD subtype and sex.

**Methods:**

This population-based study included 900 adults diagnosed with ADHD. ADHD severity, comorbidities, and functional outcomes were assessed using validated tools. Bivariate analyses and General Linear Models (GLMs) were applied to examine sex- and subtype-specific effects and their interactions.

**Results:**

Females exhibited greater ADHD severity (*p* < 0.001), higher levels of depression (*p* = 0.003) and anxiety (*p* < 0.001), lower substance use (*p* < 0.001), poorer functioning (*p* = 0.039), and greater disability (*p* = 0.001) than males. No significant sex differences were found in ADHD subtype distribution or age of symptom onset; however, females were diagnosed with ADHD later than males (*p* < 0.001). The combined ADHD subtype was associated with greater clinical severity, higher levels of depression, anxiety, and impulsive symptoms, increased substance use, and greater disability. A significant interaction between sex and subtype was observed only for disability, with females in the combined subtype exhibiting the most pronounced impairment.

**Conclusions:**

ADHD presents differently across sexes and subtypes, with specific interactions influencing disability. These findings emphasize the importance of considering sex and ADHD subtype independently to enhance diagnostic accuracy and develop targeted treatment strategies.

## Introduction

Attention-deficit/hyperactivity disorder (ADHD) is a well-known neurodevelopmental disorder that follows a clinical course through adulthood in most cases. More specifically, it is estimated that approximately two-thirds of children with ADHD will exhibit symptoms in adulthood [1]. Over the past decades, a rising prevalence of adolescent mental disorders, including ADHD and internalizing disorders, has been widely reported in the Western world [2], primarily driven by a major increase in ADHD diagnoses in adults. ADHD is relatively common in adults, with a pooled prevalence of 3.10% (95% CI 2.60–3.60%) [3]. ADHD is recognized by the clinical onset of inattention and/or hyperactivity-impulsivity symptoms, which have a remarkable impact on the lives of the people who suffer from the disorder [4]. In this regard, although ADHD is considered a childhood disorder, its symptomatic manifestations tend to become less specific and exhibit certain variations over time. More specifically, in adulthood, inattentive symptoms are more prevalent and have a greater impact on individuals’ functioning compared to hyperactive symptoms, which, by contrast, tend to cause greater impairment in children [5].

ADHD presents neurobiological, clinical, and etiological heterogeneity, which has posed a challenge in defining its diagnostic criteria over the years. To address this, the DSM-5 establishes three primary presentations of ADHD, which may vary over a person’s lifespan and are classified as predominantly inattentive, predominantly hyperactive-impulsive, and combined [6]. Moreover, the combined presentation of ADHD, characterized by the presence of both inattentive and hyperactive-impulsive symptoms, is widely recognized as the subtype with the most severe clinical impairment. In particular, research indicates how the individuals affected by this subtype experience higher levels of comorbid conditions, greater behavioral disturbances, and poorer long-term outcomes [7]. Studies indicate that this ADHD subtype’s impairments persist into adulthood, underscoring its chronic impact on quality of life and functionality [2]. Additionally, the combined presentation is associated with greater functional impairment across academic, social, and occupational domains compared to other ADHD presentations [8].

Concerning sex differences, it has been considered that ADHD predominantly affects males over females [9]. More specifically, recent literature suggests that the prevalence may be approximately twice as high in boys compared to girls [10], with this difference narrowing in adulthood, where the male-to-female ratio is approximately 1.6:1 [11]. In relation to ADHD presentations, the inattentive presentation, being less externalizing than other presentations, is suggested to be the primary factor contributing to the underdiagnosis of ADHD in female individuals. In this sense, males, who predominantly exhibit the hyperactive and combined presentation of the disorder, tend to be more aggressive and disruptive in their close environment, leading to higher rates of seeking medical advice [12, 13]. In fact, females who exhibit higher levels of hyperactivity/impulsivity also tend to present behavioral problems that facilitate early recognition of the disorder [14]. In addition to this symptomatic heterogeneity, cognitive functioning differences have also been observed between the sexes. Specifically, it has been found that while male individuals exhibit greater impulsivity and slower processing speed, female individuals show poorer outcomes in working memory and spatial reasoning [15, 16].

A recent population study has shown that the coexistence of a mental health disorder is extremely common, with approximately 80% of adult patients with ADHD estimated to have a comorbid mental health disorder [17, 18]. Several studies have shown increased severity of depression and anxiety in females, as well as lower self-esteem [19]. Specifically, females with ADHD who have been tracked into adulthood are 2.4 times more likely to be admitted to psychiatric facilities as adults compared to males with ADHD [20]. However, a notable percentage of cases of females seeking psychiatric care for a mood disorder might have undiagnosed ADHD. Indeed, in many cases, patients receive treatment for anxiety or depression while ADHD remains undiagnosed, leading to suboptimal therapeutic responses [21]. Nevertheless, when considering psychiatric comorbidity, it is known that certain disorders, such as substance use disorders, are more common among men with ADHD than among women with ADHD [22]. Globally, individuals affected by ADHD are estimated to have higher mortality rates, with this increase being more pronounced in females compared to males [23].

This study aims to analyze the differences in the severity of the disorder, its comorbidity with other psychiatric diseases, and the socio-functional impact, depending on sex and ADHD subtype. Moreover, the interaction between ADHD presentation and sex in the outcomes obtained is examined. By addressing these aspects, this study seeks to enhance the understanding of ADHD and contribute to the development of more targeted management strategies and support tailored to diverse patient populations.

## Material and methods

### Design of the study and participants

This research was conducted within the Adult ADHD Program at the Psychiatry Department of Vall d’Hebron University Hospital in Barcelona. The study received approval from the Clinical Research Ethics Committee of the Hospital Universitari Vall d’Hebron (PR(AG)103/2019). All participants voluntarily chose to take part in the study and did not receive any financial compensation.

The inclusion criteria required participants to be over 18 years old, meet the DSM-5 criteria ADHD, and agree to and sign the informed consent form before participation. Exclusion criteria included an IQ < 70; schizophrenia or other psychotic disorders; ADHD symptoms attributable to mood, anxiety, dissociative, or personality disorders; a history of adoption, sexual, or physical abuse; birth weight <1.5 kg; and other neurological or systemic disorders that might explain ADHD symptoms.

A total of 900 patients met the inclusion criteria (54.9% of whom were male, with a mean age of 36.94 ± 11.93 years). The combined presentation of ADHD was the most prevalent subtype, present in 48.2% of the participants.

### Instruments and variables

#### ADHD diagnosis

Patients with ADHD are characterized by alterations in various executive functions, with dysfunction reflected in different tests and scales. However, there is currently no test or combination of tests with sufficient positive predictive value to establish the diagnosis on an individual basis [24]. For this reason, in this study, we used the Conners Adult ADHD Diagnostic Interview for DSM-IV (CAADID-I) and the Diagnostic Interview for ADHD in Adults, Fifth Edition (DIVA-5), two of the diagnostic tools that currently show the highest sensitivity and specificity for establishing the diagnosis of this disorder [25, 26].

These instruments, validated for the Spanish population, were used to assess and confirm ADHD diagnosis. They exhibit good psychometric properties in line with DSM-5 criteria [27, 28].

#### ADHD severity

The ADHD Rating Scale (ADHD-RS) was used to assess the clinical severity of ADHD. It is a self-administered 18-item scale evaluates symptoms of attention deficit, hyperactivity, and impulsivity in adults with ADHD, and provides a high sensitivity and specificity [29]. Additionally, the Wender Utah Rating Scale (WURS) and the Clinical Global Impression Severity Scale (CGI-S) were used to assess the severity of ADHD. Both instruments are also reliable tools for evaluating these parameters [30, 31].

#### Psychiatric comorbidities and psychological characteristics

To assess psychiatric comorbidities associated with ADHD, a series of scales and tests have been used.

Firstly, the Beck-II Depression Inventory II (BDI-II) was employed to assess depressive symptoms [32]. This instrument is of significant relevance, demonstrating strong psychometric properties [33]. Secondly, the State–Trait Anxiety Inventory (STAI) was used to evaluate anxiety in terms of both state and trait components[34]. It is a widely used scale with demonstrated accuracy in both clinical and research settings [35]. Furthermore, the Barratt Impulsiveness Scale (BIS-11) was employed to assess trait impulsivity. This scale measures impulsivity as a multidimensional construct (cognitive impulsivity, motor impulsivity, and non-planning impulsivity) with robust reliability [36].

Another aspect assessed among study participants was their sleep quality, evaluated using the Pittsburgh Sleep Quality Index (PSQI). This self-reported questionnaire comprises 19 items and measures seven key aspects of sleep: sleep quality, sleep latency, sleep duration, habitual sleep efficiency, sleep disturbance, use of sleeping medication, and daytime dysfunction [37]. Currently, the PSQI stands out as one of the main standardized clinical tools encompassing a comprehensive array of indicators pertinent to sleep quality [38].

Moreover, to assess the impact of ADHD on patients’ psychosocial functioning, we utilized the Functioning Assessment Short Test (FAST). This questionnaire, consisting of 24 items distributed across six functional categories, presents evidence that supports this tool’s scientific validation and reliability in clinical practice, confirming its effectiveness in gathering comprehensive information on symptoms and functioning related to ADHD patient behavioral issues during interviews [39].

Furthermore, to assess the functional impact of the disorder, we used the World Health Organization Disability Assessment Schedule version 2.0 (WHODAS), a questionnaire that measures the individual’s level of disability across six life domains: cognition, mobility, self-care, getting along, life activities, and participation in society [40]. This tool exhibits high internal consistency and robust psychometric properties for measuring functional impairment in individuals with various health conditions, including mental health disorders [41].

Finally, to ensure a rigorous diagnostic approach, the Structured Clinical Interview for DSM-IV Axis I and II Disorders (SCID-I and SCID-II) was conducted.

### Procedure

The Adult ADHD Program at the Hospital Universitari Vall d’Hebron is a multidisciplinary and comprehensive program that evaluates and treats patients referred from primary care centers, community mental health centers, and addiction treatment units. Upon referral to the ADHD Program, patients undergo a thorough assessment to establish their diagnosis and treatment plan.

For this study, the evaluation process involved five visits conducted by trained staff specializing in ADHD, including psychiatrists and psychologists. During the initial visit, the psychiatrist conducted a comprehensive medical history, gathered pertinent sociodemographic data, and obtained relevant information regarding the patient’s ADHD clinical presentation. Alcohol and substance use were evaluated through a clinical interview conducted by trained clinicians. Participants were asked about their history of substance use, including the age of first use, age of last use, period of maximum consumption, and quantity of use. Standardized assessments using validated instruments for ADHD (CAADID-I, DIVA-5, CGI-S) were also performed. In three subsequent visits, the psychologist conducted evaluations using the following tests: WURS, ADHS-RS, BDI, STAI, BIS-11, PSQI, FAST, and WHODAS. During the fifth visit, the psychiatrist established the diagnosis of ADHD based on study findings and in accordance with DSM-5 criteria. Additionally, to ensure a rigorous diagnostic approach, the Structured Clinical Interview for DSM-IV Axis I and II Disorders (SCID-I and SCID-II) was conducted. This structured interview allowed for the confirmation of the ADHD diagnosis and the exclusion of other psychiatric disorders. Finally, regardless of whether the patient was diagnosed with ADHD, he was offered subsequent psychiatric and psychological treatment (either within our program or another unit) based on their clinical condition.

### Statistical analyses

Bivariate analyses were executed using SPSS version 26 for Windows. Initially, a comprehensive descriptive analysis of all variables was performed, including their percentages, means, and standard deviations.

Categorical variables were examined using the chi-square test, and the effect size was calculated using Cohen’s d for continuous data or Cramér’s V for nominal data. The interaction between sex and ADHD type was assessed for clinical, sociodemographic, and psychosocial functioning variables using a General Linear Model (GLM). All statistical hypotheses were tested using two-sided tests, with a p-value of less than 0.05 deemed statistically significant.

## Results

### Sex-based differences in ADHD

Sociodemographic, clinical, and functional variables are presented in Table 1. Regarding sociodemographic characteristics, no significant differences were observed between males and females, except for age and legal problems, as males were significantly younger than females (*t* = 3.673, *p* < 0.001) and had a higher incidence of legal problems (X^2^ = 24.924, *p* < 0.001).

**Table 1.** Clinical and sociodemographic characteristics of ADHD patients included in the study according to sex

	Females (*n* = 406)	Males (*n* = 494)	*t* o *X*^2^	*p*	Effect size
Sociodemographic features					
Age	39.92 ± 12.27	36.94 ± 11.93	3.673	**<0.001**	0.246
Marital status (single)	236 (62.1)	315 (67.2)	5.632	0.060	0.081
Coexistence (family)	317 (83.9)	385 (82.6)	3.018	0.221	0.060
Completed secondary education	63 (16.5)	99 (20.8)	5.001	0.082	0.076
Working/studying	300 (80)	375 (80.4)	1.842	0.765	0.047
Legal Problems: yes (%)	25 (6.6)	84 (18.1)	24.924	**<0.001**	0.172
Clinical characteristics					
ADHD (combined type)	195 (48.0)	239 (48.4)	0.011	0.485	0.003
Age of onset of dysfunctional ADHD symptoms	14.49 ± 12.17	12.53 ± 11.42	1.229	0.220	0.166
Age of ADHD diagnosis	28.96 ± 14.75	24.13 ± 15.12	3.540	**<0.001**	0.323
WURS	54.33 ± 21.86	53.06 ± 22.68	0.814	0.416	0.057
ADHD-RS	31.38 ± 8.26	29.16 ± 8.24	4.009	**<0.001**	0.269
CGI	4.05 ± 0.77	3.97 ± 0.79	1.288	0.198	0.103
Depressive symptoms (BDI)	18.61 ± 11.09	16.34 ± 10.92	2.982	**0.003**	0.206
Anxiety trait (STAI)	31.30 ± 13.05	28.94 ± 13.25	2.586	**0.010**	0.179
Anxiety state (STAI)	35.93 ± 11.34	32.50 ± 11.35	4.341	**<0.001**	0.302
Impulsivity (BIS–11)	69.57 ± 14.72	68.75 ± 16.06	0.761	0.447	0.053
Sleepiness scale (PSQI)	8.42 ± 4.87	8.14 ± 5.32	0.604	0.546	0.055
FAST	27.45 ± 10.82	25.78 ± 11.70	2.064	**0.039**	0.148
WHODAS	31.51 ± 10.72	28.91 ± 10.10	3.287	**0.001**	0.538
Substance use					
Alcohol	114 (28.2)	194 (39.4)	12.364	**<0.001**	0.117
Tobacco	107 (26.6)	161 (32.7)	4.024	**0.026**	0.067
Cannabis	52 (12.9)	111 (22.6)	13.872	**<0.001**	0.124

Abbreviations: WURS, Wender Utah Rating Scale; ADHD-RS, ADHD Rating Scale; CGI, Clinical Global Impression Scale; BDI, Beck-II Depression Inventory II; STAI, State–Trait Anxiety inventory; BIS-11, Barratt Impulsiveness Scale; PSQI, Pittsburgh Sleep Quality Index; FAST, Functioning Assessment Short Test; WHODAS, World Health Organization Disability Assessment Schedule 2.0. Significant differences (*p* < 0.05) marked in bold.

**Figure 1.**
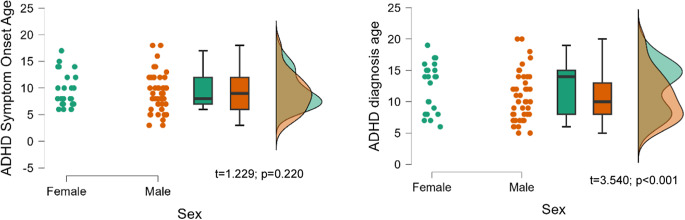
Age of onset of dysfunctional ADHD symptoms and diagnosis by sex.

No significant differences were found in the age of onset of dysfunctional ADHD symptoms (*p* = 0.220). However, a later age of ADHD diagnosis was observed in females (28.96 ± 14.75 years versus 24.13 ± 15.12 years, *t* = 3.540, *p* < 0.001) (see Figure 1).

Regarding the severity of ADHD, females exhibited higher scores on the ADHD-RS (*t* = 4.009, *p* < 0.001). As expected, female patients with ADHD were more likely to exhibit higher levels of depression and anxiety than male patients, as reflected by their higher scores on the BDI (*p* = 0.003) and STAI scales (trait, *p* = 0.010; state, *p* < 0.001). However, no statistically significant differences were observed between females and males in terms of impulsivity and insomnia. The females included in the study also exhibited greater impairment in psychosocial functioning and disability, obtaining higher scores on the FAST (*t* = 2.064, *p* = 0.039) and WHODAS (*t* = 3.287, *p* = 0.001) tests compared to the males.

Finally, males exhibited higher rates of substance use compared to females. Specifically, alcohol use was reported by a greater percentage of males (39.4%) than females (28.2%) (X^2^ = 12.364, *p* < 0.001). Similarly, tobacco use was more prevalent among males (32.7%) than females (26.6%) (X^2^ = 4.024, *p* = 0.026). Cannabis use followed this pattern, with 22.6% of males reporting use compared to 12.9% of females (X^2^ = 13.872, *p* < 0.001).

### Differences across ADHD subtypes

Table 2 presents an analysis of the sociodemographic and clinical characteristics of the patients included in the study based on their ADHD subtype. A higher proportion of participants with the inattentive subtype were working or studying (82.6%) compared to those with the combined subtype (77.7%, *p* = 0.042). Patients with the combined subtype reported more legal problems (17.7% versus 8.6%, *p* < 0.001).

**Table 2.** Clinical and sociodemographic characteristics of ADHD patients included in the study according to ADHD type

	Combined ADHD (*n* = 434)	Inattentive ADHD (*n* = 466)	*t* o *X*^2^	*p*	Effect size
Sociodemographic features					
Age	39.57 ± 12.08	37.02 ± 12.15	3.166	**0.002**	0.210
Sex (female)	195 (44.9)	211 (45.3)	0.011	0.485	0.003
Marital status (single)	250 (61.7)	301 (67.8)	3.680	0.159	0.066
Coexistence (family)	328 (81.2)	374 (85)	2.331	0.312	0.053
Completed secondary education	81 (19.9)	81 (18)	5.574	0.062	0.081
Working/studying	313 (77.7)	362 (82.6)	3.287	**0.042**	0.063
Legal problems: yes (%)	71 (17.7)	38 (8.6)	5.380	**<0.001**	0.153
Clinical characteristics					
Age of onset of dysfunctional ADHD symptoms	13.41 ± 12.61	13.56 ± 10.80	−0.092	0.927	0.013
Age of ADHD diagnosis	26.18 ± 15.32	25.95 ± 15.03	0.165	0.869	0.015
WURS	57.36 ± 23.65	49.95 ± 20.28	4.842	**<0.001**	0.336
ADHD-RS	32.20 ± 8.20	28.13 ± 7.97	7.542	**<0.001**	0.503
CGI	4.18 ± 0.77	3.85 ± 0.76	5.658	**<0.001**	0.431
Depressive symptoms (BDI)	18.40 ± 11.24	16.42 ± 10.74	2.624	**0.009**	0.180
Anxiety trait (STAI)	31.42 ± 13.30	28.65 ± 12.95	3.061	**0.002**	0.211
Anxiety state (STAI)	35.11 ± 11.72	33.01 ± 11.14	2.656	**0.008**	0.184
Impulsivity (BIS–11)	73.24 ± 14.10	65.02 ± 15.64	7.968	**<0.001**	0.552
Sleepiness scale (PSQI)	8.28 ± 5.47	8.28 ± 4.81	−0.004	0.997	0
FAST	26.97 ± 11.62	26.07 ± 11.00	1.116	0.265	0.080
WHODAS	31.28 ± 10.77	28.92 ± 10.00	3.009	**0.003**	0.227
Substance use					
Alcohol	170 (39.4)	138 (29.7)	9.456	**0.002**	0.103
Tobacco	152 (35.3)	116 (25.0)	11.228	**0.001**	0.112
Cannabis	106 (24.6)	57 (12.3)	22.730	**<0.001**	0.159

Abbreviations: WURS, Wender Utah Rating Scale; ADHD-RS, ADHD Rating Scale; CGI, Clinical Global Impression Scale; BDI, Beck-II Depression Inventory II; STAI, State–Trait Anxiety Inventory; BIS-11, Barratt Impulsiveness Scale; PSQI, Pittsburgh Sleep Quality Index; FAST, Functioning Assessment Short Test; WHODAS, World Health Organization Disability Assessment Schedule 2.0. Significant differences (*p* < 0.05) marked in bold.

Results indicate that patients with a combined presentation of ADHD exhibited a higher severity of the disorder (as measured by the WURS, ADHD-RS, and CGI scales) compared to those with the inattentive presentation. Additionally, patients with the combined subtype reported significantly higher levels of depression (BDI), anxiety (STAI), and impulsivity (BIS-11), while no differences were observed in sleep quality (PSQI). Participants diagnosed with the combined presentation of ADHD reported higher levels of disability (as WHODAS) compared to those with the inattentive presentation; however, no significant differences were observed in psychosocial functioning (FAST) between the two groups.

Finally, substance use patterns also differed between subtypes. Participants with the combined presentation showed higher rates of alcohol consumption (39.4% versus 29.7%, *p* = 0.002), tobacco use (35.3% versus 25.0%, *p* = 0.001), and cannabis use (24.6% versus 12.3%, *p* < 0.001) compared to those with the inattentive subtype.

### Interaction between sex and ADHD subtypes

**Figure 2.**
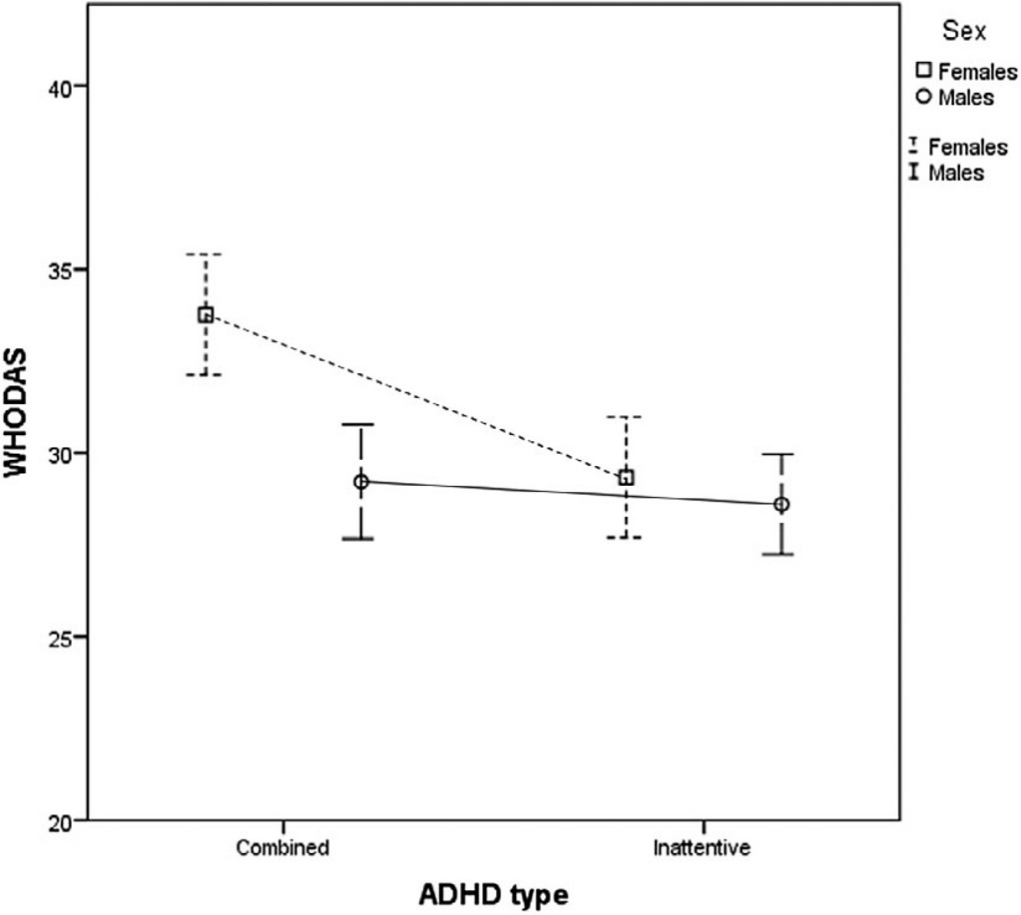
Interaction between sex and subtype of ADHD on functional impact as measured by the WHODAS scale.

Despite observing differences in symptom severity and functional impairment depending on sex or ADHD subtype, the interaction between sex and ADHD subtype did not significantly influence these outcomes (Table 3), except for disability (WHODAS, *p* = 0.015) (Figure 2). Specifically, females with the combined ADHD subtype exhibited greater disability compared to both males with the same subtype and individuals of either sex with the inattentive subtype.

**Table 3.** Clinical characteristics according to ADHD subtype and sex

	Combined ADHD (*n* = 434)		Inattentive ADHD (*n* = 466)		Sex * ADHD type	
	Females	Males	Females	Males	*X*^2^	*p*
Clinical characteristics						
Age of onset of dysfunctional ADHD symptoms	13.20 ± 1.51	13.62 ± 1.49	16.17 ± 1.72	11.25 ± 1.62	2.84	0.092
Age of ADHD diagnosis	29.40 ± 1.65	24.22 ± 1.29	28.65 ± 1.38	24.06 ± 1.15	0.05	0.831
WURS	59.44 ± 1.65	55.85 ± 1.49	49.52 ± 1.60	50.45 ± 1.43	2.15	0.143
ADHD-RS	33.73 ± 0.58	31.08 ± 0.52	29.25 ± 0.55	27.33 ± 0.50	0.46	0.495
CGI	4.18 ± 0.06	4.19 ± 0.06	3.93 ± 0.06	3.78 ± 0.06	1.60	0.205
Depressive symptoms (BDI)	19.79 ± 0.80	17.18 ± 0.73	17.48 ± 0.78	15.54 ± 0.71	0.20	0.653
Anxiety trait (STAI)	32.98 ± 0.96	30.05 ± 0.88	29.67 ± 0.94	27.88 ± 0.85	0.40	0.530
Anxiety state (STAI)	37.45 ± 0.83	33.25 ± 0.76	34.48 ± 0.81	31.78 ± 0.74	0.89	0.346
Impulsivity (BIS–11)	74.02 ± 1.10	72.81 ± 1.01	65.41 ± 1.07	64.89 ± 0.98	0.11	0.740
Sleepiness scale (PSQI)	7.96 ± 0.55	8.50 ± 0.48	8.73 ± 0.45	7.85 ± 0.43	2.19	0.139
FAST	28.33 ± 0.86	25.89 ± 0.79	26.65 ± 0.82	25.68 ± 0.76	0.84	0.361
WHODAS	33.77 ± 0.82	29.22 ± 0.75	29.34 ± 0.80	28.61 ± 0.75	5.97	**0.015**

Abbreviations: WURS, Wender Utah Rating Scale; ADHD-RS, ADHD Rating Scale; CGI, Clinical Global Impression Scale; BDI, Beck-II Depression Inventory II; STAI, State–Trait Anxiety Inventory; BIS-11, Barratt Impulsiveness Scale; PSQI, Pittsburgh Sleep Quality Index; FAST, Functioning Assessment Short Test; WHODAS, World Health Organization Disability Assessment Schedule 2.0. Significant interactions (*p* < 0.05) marked in bold.

## Discussion

The current study provides new insights into the clinical severity of ADHD and its impact, with a focus on differences between males and females and ADHD subtypes.

One of the most significant findings in the studied sample is the later age at which ADHD is diagnosed in females compared to males. This finding is consistent with the fact that male individuals more frequently present the hyperactive/impulsive variant of the disorder and are thus more likely to be referred to a specialist at an earlier age, allowing for earlier recognition of the disorder [42]. Conversely, and in line with findings from other studies, female individuals diagnosed with ADHD exhibit greater clinical severity of the disorder. This may be related to the higher likelihood of missed or delayed diagnosis in women, which ultimately leads to a greater impact of the disorder in affected females [16].

Consistent with a recently published meta-analysis [43], female individuals were found to be more affected by anxiety and depression than male individuals. However, this relationship does not extend to other disorders examined, as our results indicate that sex does not significantly influence the risk of increased impulsivity or poorer sleep quality. Regarding psychosocial functioning impairment, our findings align with current evidence, showing greater difficulties in females. These results may suggest that late detection of the disorder, higher psychiatric comorbidity, and delays in initiating accurate treatment for female patients with ADHD contribute to greater social difficulties and feelings of rejection by peers from an early age [44, 45]. Furthermore, this study, in line with recent literature, has demonstrated that adult males with ADHD have a higher prevalence of other psychiatric disorders, such as substance use disorders or disruptive behavior disorders, compared to adult females [46].

When examining the severity of ADHD based on subtype, we found that individuals with the combined presentation scored higher on the WURS scale, which measures the intensity of symptoms reported in childhood, and on the ADHD-RS scale, which evaluates current symptomatology. These findings are consistent with a German study that also utilized the WURS scale and reported higher scores for individuals with the combined subtype [47]. The greater clinical impact of the combined presentation is likely attributable to the higher prevalence of disruptive behaviors and psychiatric comorbidities associated with this subtype, as highlighted by a Chinese study [48]. Regarding functional outcomes, our findings indicate that individuals with the combined presentation exhibit significantly greater levels of disability (WHODAS), but no differences were observed in psychosocial functioning (FAST). The combined subtype’s higher rates of impulsivity, substance use, and legal problems may exacerbate perceived disability across multiple life domains, which could explain the observed differences in WHODAS scores. Supporting this, an Italian study [49] reported that individuals with the combined presentation exhibit greater deterioration in personal relationships, likely driven by impulsive and inappropriate behaviors. The differences observed between ADHD subtypes in WHODAS scores but not in FAST scores could be attributed to the differences in how these scales capture functional impairments. The WHODAS 2.0 provides a broader overview of disability by capturing the overall impact of functional impairments across multiple life domains. This may be particularly relevant for individuals experiencing impulsivity-driven disruptions or external consequences, such as legal issues. In contrast, FAST offers a more focused assessment of specific areas of daily functioning, such as autonomy, work, interpersonal relationships, and leisure [50].

The lack of a significant interaction between sex and ADHD subtype for most clinical and functional outcomes suggests that, while there are clear differences in symptom severity and functional impairment based on sex and ADHD subtype, these factors largely operate independently. In other words, both male and female participants exhibit similar patterns of symptom severity within each ADHD subtype. However, it is important to note that females with the combined ADHD subtype demonstrated greater disability (WHODAS) compared to males with the same subtype and individuals of either sex with the inattentive subtype. This finding indicates that, although sex does not broadly influence symptom severity within ADHD subtypes, it plays a specific role in modulating disability in individuals with the combined presentation. These results highlight the importance of considering both sex and ADHD subtype independently when assessing and treating individuals with ADHD. Several factors may contribute to this observed disparity in disability among females with the combined subtype. Biologically, hormonal fluctuations, particularly estrogen’s influence on dopamine regulation, may impact emotional regulation, stress response, and executive functions in ADHD [51]. Given that executive function and emotional regulation are crucial for managing daily life demands, hormonal effects may exacerbate disability in females with the combined subtype. Psychosocial factors also play a role, as women with ADHD often face greater societal expectations related to organization, multitasking, and emotional regulation, leading to increased perceived impairment when these expectations are not met [16]. Additionally, females with ADHD are diagnosed later than males, often after prolonged struggles with unrecognized symptoms [19]. The cumulative effect of delayed diagnosis and inadequate intervention may contribute to increased disability scores. Furthermore, coping mechanisms differ by sex: males with ADHD are more likely to externalize symptoms through impulsivity and risk-taking behaviors, whereas females tend to develop compensatory strategies such as excessive self-monitoring and emotional suppression. Although these strategies may help mask symptoms in the short term, they often result in chronic stress and emotional exhaustion, further contributing to higher disability scores [16, 19]. These findings highlight the importance of incorporating disability measures in ADHD assessments, particularly for females, as symptom severity scales alone may not adequately capture the full extent of the disorder’s impact on daily functioning and quality of life.

This study has several limitations that should be considered. First, the sample was drawn from a clinical setting, which may not fully represent the broader population of individuals with ADHD. This could introduce selection bias and limit the generalizability of the findings. Second, the cross-sectional design of the study precludes causal inferences between ADHD subtype, sex, and the observed clinical and functional outcomes. Longitudinal studies are needed to explore the temporal relationships between these factors. Despite these limitations, the study has notable strengths. The inclusion of a diverse cohort of participants from a specialized ADHD clinic enables a more comprehensive analysis of the disorder across different subtypes and sexes. Additionally, the large sample size enhances the statistical power and reliability of the findings. Furthermore, the use of multiple validated instruments to assess ADHD severity, psychiatric comorbidities, and functional impairment ensures a robust, multidimensional evaluation of the disorder’s impact on individuals.

In conclusion, this study underscores the complexity of ADHD as a multifaceted disorder influenced by both sex and ADHD subtype. While our findings indicate that sex does not broadly influence the relationship between ADHD subtype and symptom severity, it may have a more nuanced impact on specific aspects of disability, particularly in females with the combined presentation. These insights are crucial for improving diagnostic accuracy and developing more tailored treatment strategies. Future research should continue to explore these dimensions, considering how other variables, such as age, socioeconomic status, comorbid conditions, sociocultural differences, and biological conditions specific to the female sex, such as the regulation of hormonal cycles, might interact with sex and subtype to influence ADHD outcomes.

## Data Availability

The data that support the findings of this study are available on request from the corresponding authors.
